# Might hydrogen peroxide reduce the hospitalization rate and complications of SARS-CoV-2 infection?

**DOI:** 10.1017/ice.2020.170

**Published:** 2020-04-22

**Authors:** Arturo A. Caruso, Antonio Del Prete, Antonio I. Lazzarino, Roberto Capaldi, Lucia Grumetto

**Affiliations:** 1 Department of Otolaryngology, AIAS Structure of Afragola, Afragola, Naples, Italy; 2 Department of Neurosciences and Reproductive and Dentistry Sciences, School of Medicine and Surgery, University of Naples Federico II, Naples, Italy; 3 EPISTATA – Agency for Clinical Research and Medical Statistics, London, United Kingdom; 4 School of Medicine and Surgery, University of Naples Federico II, Naples, Italy; 5 Pharm-Analysis and Bio-Pharm Laboratory, Department of Pharmacy, School of Medicine and Surgery, University of Naples Federico II, Naples, Italy

*To the Editor—*The severe acute respiratory syndrome coronavirus 2 (SARS-CoV-2) is the virus responsible for COVID-19, which emerged in Wuhan, China, in December 2019. The current pandemic appears to be characterized by human-to-human transmission; it occurs through cough, sneeze, droplet inhalation, and direct contact of hands with mouth, nose, and eyes. The virus resides in the mucous membranes and it is transmitted through the saliva and the respiratory droplets. Although prevention of person-to-person transmission is the key to limiting the pandemic, so far, little attention has been given to the events taking place immediately after the onset of the first symptoms.

To prevent the spread of the virus, in February 2020, the Italian government issued a recommendation, among the methods of sanitizing the environments, for the use of 0.5% hydrogen peroxide.^1^ Hydrogen peroxide is already widely used as an environmental, surgical disinfectant and as an oral disinfectant in the treatment of gingivitis.^2,3^ SARSCoV-2 is spread by human-to-human transmission; the infection is estimated to have an average incubation period of 6.4 days and a base reproduction number of 2.24–3.58.^4^ Furthermore, scientific studies have proven that the virus persists for 2 days on the mucous membranes of macaques^5^ before the subsequent spread of the virus to the lower respiratory tract. This delay represents a window of therapeutic opportunity.

The efficient inactivation of coronaviruses (eg, SARS and MERS) on inanimate surfaces using hydrogen peroxide (H_2_O_2_ 0.5% for 1 minute) was assessed by Kampf et al.^6^ Based on their findings, and after reviewing the current literature concerning hydrogen peroxide, we propose that hydrogen peroxide, as an antiseptic agent, could play a pivotal role in reducing the hospitalization rate and COVID-19–related complications. The antiseptic efficacy of hydrogen peroxide 3% against SARSCoV-2 on oral and nasal mucosa can be reasonably hypothesized. The antiseptic action is due not only to the known oxidizing and mechanical removal properties of hydrogen peroxide but also to the induction of the innate antiviral inflammatory response by overexpression of Toll-like receptor 3 (TLR3).,^7^ Thus, the overall progression of the infection from the upper to the lower respiratory tract can be reduced.

Therefore, we advise an off-label use of H_2_O_2_ 3% and 1.5 % (10 volumes) by oral and nasal washing respectively, performed immediately after the onset of the first symptoms and the presumptive diagnosis of COVID-19 and during the illness in home quarantine or by hospitalized patients not requiring intensive care.

**Fig. 1.**
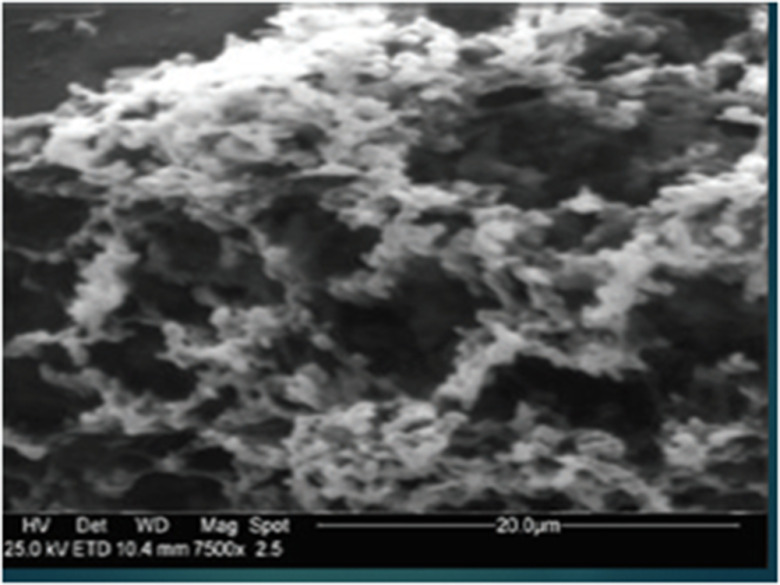
Mouth mucous membranes after administration of H_2_O_2_ 3% (10 vol) over a period of six months (Scraping cytology and scanning electron microscopy; SEM 7500 Cambridge MARK 250 SEM).

We propose a regimen of gargling 3 times per day for disinfection of the oral cavity and nasal washes with a nebulizer twice daily (due to a greater sensitivity of the nasal mucosa). Hydrogen peroxide (H_2_O_2_) is safe for use on the mucous membranes as gargling or as a nasal spray; in fact, it is already commonly used in otolaryngology. Figure 1 shows the epithelial of oral mucosa treated with H_2_O_2_ 3% for a period of 6 months. No damage was observed on oral mucous membranes or their microvilli after ongoing gargling treatment with H_2_O_2_ 3%. Another route for SARSCoV-2 is through nasolacrimal ducts; thus, we advise the use of iodopovidone 0.5%–0.6% as eye drops (1 drop 3 times daily on conjunctiva of both eyes) due to its antiseptic action against SARS-CoV-2 within 1 minute.

In our opinion, the effectiveness of this regimen will be verified through a significant reduction of the rate of hospitalization and respiratory complications in patients positive for SARS-CoV-2 with and without mild-to-moderate symptoms. We strongly encourage the rapid development of randomized controlled trials including both SARS-CoV-2–positive and –negative participants to study the benefits of H_2_O_2_ 3% in the reduction of pulmonary complications and hospitalization rates.
